# Association between constitution, axiography, orthodontic cast analysis, and upper body posture in women aged 31 to 40 years

**DOI:** 10.1007/s00784-023-05030-1

**Published:** 2023-05-01

**Authors:** C. Loewe, J. Pflaum, E. M. Wanke, C. Erbe, F. Holzgreve, D. A. Groneberg, Daniela Ohlendorf

**Affiliations:** 1 Institute of Occupational Medicine, Social Medicine and Environmental Medicine, Goethe-University Frankfurt Am Main, Theodor-Stern-Kai 7, Building 9A, 60590 Frankfurt Am Main, Germany; 2 Department of Orthodontics, School of Dentistry, University Medical Center of the Johannes Gutenberg University Mainz, Augustusplatz 2, 55131 Mainz, Germany

**Keywords:** Women, Axiography, Dental casts, Upper body posture, Constitution

## Abstract

**Objective:**

Whether it is primarily the spine that reacts with pain to the negative consequences of everyday stress and possibly the temporomandibular system as a result (ascending chain), or whether incorrect stress in the dental area has an influence on body geometry (descending chain), is still a controversially discussed topic. The aim of this study is to investigate possible relationships between constitutional, axiographic, and dental parameters with upper body posture.

**Material and methods:**

A total of 106 subjectively healthy women between 31 and 40 years of age voluntarily participated in this study. Data collection was done by filling out a questionnaire with constitutional and anamnestic parameters and by evaluating orthodontic casts, axiographic measurements, and video raster stereographic measurements. These data were analyzed using correlations and group comparisons, with the significance level set at *p* ≤ 0.05.

**Results:**

Positive correlations were shown between the constitutional factors of body weight and BMI and the lumbar bending angle (*p* = 0.01), the kyphosis angle (*p* = 0.001), and lordosis angle (weight *p* = 0.05; BMI *p* = 0.03). In the cast analysis, regardless of the direction of the midline shift (left/right/none), a left lateral tilt can be seen which is greatest at 2.12° with a left midline shift. In addition, the elevated pelvic side correlates with the side of the displacement of the jaw, with the stronger manifestation being on the left side. With a vertical anterior bite in the normal range, the kyphosis angle is 48.09°, while with a deep bite, it is 60.92°, and with an open bite, it is 62.47°; thus, the group in the normal range differs significantly (*p* = 0.01) from the other two. The greater the protrusion, the smaller the sagittal plane angles (kyphosis angle, lumbar bending angle, each *p* = 0.03), and the more dorsal the posture (*p* = 0.04). The lordosis angle differs significantly (*p* = 0.001) between the group of subjects with a protrusion in the normal range (52.34°) and the group with an increased advancement of the mandible (41.79°).

**Conclusion:**

There is a correlation between body weight, BMI, midline shift, and protrusion, as well as the vertical anterior step and upper body posture in women between 31 and 40 years of age. Interdisciplinary functional examinations of the temporomandibular musculature, and also sustained orthodontic treatment, can contribute to an improvement in upper body posture.

## Introduction

Physiologically balanced upper body posture is beneficial for pain-free postural and movement control. In Germany, 22.5% of the population between the ages of 20 and 75 years suffer from back pain, one of the main causes of incapacity to work [1]. In this context, occupational stress to perform in western industrial societies is reflected in not only the increasing pain symptoms of the spine, but also the increase in harmful habits such as teeth grinding and clenching, which sometimes lead to serious consequences in the temporomandibular joint, neck, and head areas (e.g., temporomandibular dysfunction) [2, 3]. Whether it is primarily the spine that reacts with pain to the negative consequences of everyday stress, and possibly the temporomandibular system as a result (ascending chain) or vice versa (descending chain), is a controversial topic and remains of great importance [4–7].

In this context, numerous studies have been conducted [5–29]. Gadotti et al. [8] found a more anteriorly directed head posture in patients with Angle class II malocclusions; likewise, Nobile et al. [9] associated Angle class II with anteriorly displaced posture and class III with posteriorly displaced posture. Solow et al. [10] used cephalometric radiographs to show that children with anterior crowding had significantly larger craniocervical angles of 3–5° on average than those without crowding. Lippold et al. [11] were able to demonstrate two patterns of correlations between craniofacial morphology and the back shape profile (by means of video raster stereography) in adults: on the one hand, a more distal and vertical craniofacial pattern with a larger upper thoracic, lumbar-lordotic, and pelvic angle, and, on the other hand, a more mesial and horizontal craniofacial pattern with small upper thoracic, lumbar-lordotic, and pelvic angle. In another investigation, Lippold et al. [12] registered correlations between scoliosis or poor posture and Angle class II in 59 preschool children.

Nobili et al. [9] examined 50 subjects in various mandibular positions with the eyes open and closed using a pressure measurement plate; they found an association between an Angle class II with an anteriorly displaced posture and a class III with a posteriorly displaced posture. Klostermann et al. [13] analyzed the relationship between posture measured by video raster stereography and sagittal overbite (Angle class II) in children before and after early orthodontic treatment with removable functional orthodontic appliances. The authors demonstrate a significant reduction of the overbite, which is associated with improved posture and back parameters, such as pelvic torsion. However, these changes are mostly just above the measurement error and should therefore be assessed with caution.

In their review, Langella et al. [14] found no evidence of positive effects of orthodontic treatment on spinal deformity and criticize the quality of these studies in particular. The same is confirmed by Michelotti et al. [15]. This review also points out that there is not enough scientific evidence to prove a cause-effect relationship. A further review confirms that the existing literature is not sufficient to prove the relationship between head and cervical posture and sagittal malocclusion [16].

In accordance with this review, other authors found no significant correlations between occlusion and posture (e.g., measured with video raster stereography) [17–21].

The video raster stereography technique was also used to analyze temporary dental changes of a symmetrical occlusal block in the premolar region in terms of an ad hoc effect on upper body posture. Here, the upper body posture of healthy people changes only very slightly due to a temporary symmetrical change in the bite position without predominant age- and gender-specific reactions so that neurophysiological compensation mechanisms seem to function almost equally well [22].

Therefore, the aim of the present analysis is to examine female participants between 31 and 40 years of age regarding possible correlations between upper body posture and dental or axiographic parameters. In order to limit the results in terms of age and gender, this study exclusively examines women between 31 and 40 years of age since growth complaints, as well as changes in the sense of increasing thoracic kyphosis due to degenerative changes in the intervertebral discs and thus effects on posture [30, 31] (as in children or older people), can be excluded here. Likewise, the focus is only on one sex, namely the female, since men are fundamentally different from women in terms of physique and body composition as well as hormone balance [32, 33] or their different sensitivity with regard to temporomandibular dysfunction [34]. The present back scan data are part of a larger project [35] and were (integrated into two analysis with different research questions and have already been published [22, 23].

The following hypotheses will now be tested:

1. Increasing height, body weight, and BMI worsen upper body posture in the sagittal direction.
2. Midline shifts in the maxilla and mandible are associated with spinal and pelvic deviation in the frontal plane.
3. Protrusion and laterotrusion of the mandible correlate negatively with the values of the spine in the sagittal and frontal direction.

## Material and methods

### Subjects

A total of 106 subjectively healthy adult women (102 right-handed) aged 31 to 40 years (35.14 ± 2.98 years) voluntarily participated in the present study.

“Subjectively healthy” means that each woman can negate the following exclusion criteria: medically diagnosed malpositions as well as acute pain of the spine, shoulders, or pelvis as well as acute pain in the temporomandibular system, operations on/accidents to the musculoskeletal system or the temporomandibular system in the last 2 years, and use of muscle relaxants. Furthermore, the participants were not allowed to wear removable dentures and had to have a clear occlusion in order to perform an accurate cast analysis. Thus, a participant who is slightly overweight by WHO definition [36] can describe herself as healthy if she does not meet any of the above exclusion criteria. Moderate musculoskeletal complaints were acceptable as long as they did not affect the participant’s daily life.

The average height of the participants was 1.66 ± 0.06 m, body weight 67.15 ± 13.39 kg, and BMI 24.25 ± 4.65 kg/m^2^ (height and weight based on subjective information). According to the WHO classification [36], 67 women were normal weight (group II; 18.50–24.90 kg/m^2^), 25 overweight (group III; 25–29.90 kg/m^2^), and 11 obese (group IV; ≥ 30 kg/m^2^). Only 3 of the 106 participants were underweight (group I; BMI < 18.50 kg/m^2^). Orthodontically completed treatment was present in 43 (40.57%) of the 106 subjects.

Regarding the Angle class distribution, 27.36% (29 women) had Angle class I, 37.74% (40 women) had Angle class II, and 34.90% (37 women) had Angle class III malocclusions.

Eighty women (75.47%) had a predominantly sedentary occupation (e.g., office clerk), 16 women (15.09%) reported mainly standing (e.g., production worker), and 10 participants (9.43%) had considerable movement in their occupation (e.g., waitress).

Before the commencement of the study, each participant signed a written consent to participate voluntarily and filled out a medical history form [37]. This was used to determine the exclusion criteria from the study.

An approved ethics application of the medical faculty of the Goethe-university Frankfurt/Main (ethics no. 103/16) is available for the present study. The requirements of the ethics application are based on the ethical principles that apply to medical research on living subjects (humans). The principles are published in the latest edition of the Declaration of Helsinki, Finland (2013).

### Measuring systems

#### Situation impression and orthodontic cast analysis

The modeling wax “Standard” (Steinhart, Krumbach/Germany) was used for a squeeze bite. Afterwards, situational impressions of the upper and lower jaw were taken. The alginates “Omnicolor” (Omnident, Rodgau Nieder-Roden/Germany) and “HS-Alginate” (Henry-Schein Dental, Frankfurt/Germany) were used for this purpose. The impressions were cast with the hard plaster “Natura” (Siladent, Goslau/Germany) in order to carry out an orthodontic cast analysis according to Schopf [38]. For this purpose, the plaster casts were measured with the caliper “Münchener Modell” (Smiledental, Düsseldorf/Germany). The following dental parameters were recorded: transversal dental arch width maxilla and mandible, horizontal and vertical anterior step, midline shift in the mandible, occlusion of the first molars on the right and left, and the angle classes.

#### Axiography

The mandibular movements of the study participants were recorded with the help of an axiograph (JMAnalyser, Zebris Medical GmbH, Isny im Allgäu/Germany) of the type BT2. The axiograph works on the basis of a 3D ultrasound navigator with a frequency of 50 kHz [39]. Based on the runtime between the transmitters and receiver microphones, 16 measurement distances result from which the absolute spatial coordinates of the geometrically defined positions are calculated using the triangulation theory. The accuracy in the occlusal area is ± 0.1 mm/ ± 2° [39, 40]. In this way, discoordination of the occlusion or the temporomandibular joint, such as movement restrictions or hypermobility, can be revealed.

The following movements were performed: laterotrusion left/right, protrusion, maximum mouth opening, deviation, and deflection.

#### Three-dimensional back scanner

The “ABW Mapper mobile” (ABW GmbH, Frickenhausen/Germany) is used for contactless and radiation-free three-dimensional measurement of the back. For a three-dimensional image of the back surface, 30 video images are recorded with a maximum possible frame rate of 50 images per second. The three-dimensional result image is displayed with a spatial depth resolution of 1/100 mm. The manufacturer states that the measurement error is less than 1 mm. Repeat measurements result in a reproducibility of less than 0.5 mm.

A projector is integrated into this device which projects a stripe pattern onto the surface of the spine. An LCD camera (600 × 400 mm, resolution 640 × 480 pixels) records the pattern of the light projection from a defined angle so that further evaluations can be carried out using triangulation technology. Thus, the surface of the back, starting from the seventh cervical vertebra up to the rima ani, is recorded three-dimensionally by light optometry and it is possible to assess the shoulder and pelvic area (sagittal and frontal changes) as well as the shape of the spine (in terms of a lordotic or kyphotic posture or a scoliotic malposition).

For the assessment of the dorsal upper body posture, six previously determined anatomical fixed points on the bare back were marked with reflective markers so that the angle for the spinal, shoulder, and pelvic regions could be calculated from two markers each:

A. VP: Vertebra prominens (7th cervical vertebra)
B. AISL: Angulus inferior scapulae left (lower scapular angle left)
C. AISR: Angulus inferior scapulae right (lower shoulder blade angle right)
D. DL: Dimpel’s lumbar dimple left (spina iliaca posterior superior left) (posterior superior iliac spine left)
E. DR: Dimpled lumbar dimple right (spina iliaca posterior superior right) (posterior superior iliac spine right)
F. SP: Sacrum point (beginning of the rima ani)

Furthermore, the method of video raster stereography is found to be reliable for both intratest and intertest reliability [41–43]. In the sagittal plane and partially for scoliosis parameters, it is a reliable method for non-invasive, three-dimensional assessment of spinal alignment in normal, non-scoliotic individuals [41]. The reliability of most parameters was excellent [42] or very good for both intratester and intertester reliability [43]. The technique is well applicable for the analysis of the back in a standing position, where the body mass index has no influence on the reproducibility. Here, the scan can be used for both scientific and practical recommendations in the context of spinal screening [41].

### Examination procedure

#### Situation impression and orthodontic cast analysis

After taking the situation impression of the upper and lower jaw, a plaster cast was made in the laboratory by pouring the impressions. For the fabrication of a squeeze bite, a heated plate of modeling wax was placed between the dentition of the test person on which they bit until it hardened. This bite was taken in habitual occlusion to make it easier to assemble the casts and to trim them three-dimensionally. The finished plaster casts could then be measured with a gauge, and a cast analysis, according to Schopf (University of Frankfurt), could be carried out.

#### Axiography

In preparation for the examination, the paraocclusal attachment was first vestibularly fixed to the mandibular teeth with autopolymerizing acrylic (“LuxaBite”) (DMG, Hamburg/Germany) in order to fix the mandibular sensor to it via a magnet. In the second step, the upper jaw sensor was placed on the head. Prior to the first measurement, a “calibration” and subsequent practice runs of the jaw movements were carried out.

All jaw movements (laterotrusion left/right, protrusion, maximum mouth opening, deviation and deflection) were recorded in a sitting position, in front of the computer screen, with a straight gaze, upright head and body posture, and a relaxed shoulder and arm position. The starting and ending positions were always the habitual final bite situation.

To avoid statistical errors, these movement sequences were recorded three times each.

#### Three-dimensional back measurement

For the three-dimensional back measurement, each participant stood barefoot and unclothed in front of the back scanner at a distance of about 90 cm. To ensure the same starting position for all participants, a distance piece was placed between the scanner and the measuring position. This also ensured that the participants always stood exactly parallel to the scanner with both heels on the template. The six anatomical structures were then marked using skin-friendly, reflective adhesive dots with a diameter of 1 cm. Each subject was instructed to adopt a habitual head and body posture with a relaxed shoulder and arm position (arms hanging down) and the feet about a hip-width apart, looking straight ahead and not speaking.

At least three series of measurements in habitual occlusion were carried out for each test person in order to calculate the mean value and to reduce statistical errors.

### Statistical analysis

The statistical analysis of the data was carried out using the program BIAS version 11.06–04/2017 (epsilon Verlag, Darmstadt). The collected data were tested for normal distribution using the Kolmogorov–Smirnov-Lilliefors test. For all parameters, the mean or median, the confidence interval, and the tolerance range were determined according to their (non-)normal distribution. The two-sided 95% tolerance range and the two-sided 95% confidence interval were calculated. Furthermore, the Wilcoxon Mann–Whitney *U* test and Kruskal–Wallis test with post hoc tests (Conover-Iman comparison) were used. All *p*-values were subjected to a subsequent Bonferroni-Holm correction. The simple, linear Pearson correlation matrix or the rank correlation matrix according to Spearman and Kendall was also used. For the correlation data, the effect size was classified according to Cohen, for example, a correlation coefficient of rho = 0.1 indicated a low correlation, rho = 0.3 indicated a medium correlation, and with rho = 0.5, there was a high correlation. The significance level was *p* ≤ 0.05.

## Results

### Constitution

The left shoulder angle (*p* ≤ 0.001) becomes smaller with increasing weight or BMI, while the left shoulder is positioned further cranially. With increasing height and body weight, trunk length D (height *p* = 0.001, weight *p* ≤ 0.02) and S (both *p* ≤ 0.001) increase (Table 1).

**Table 1:**
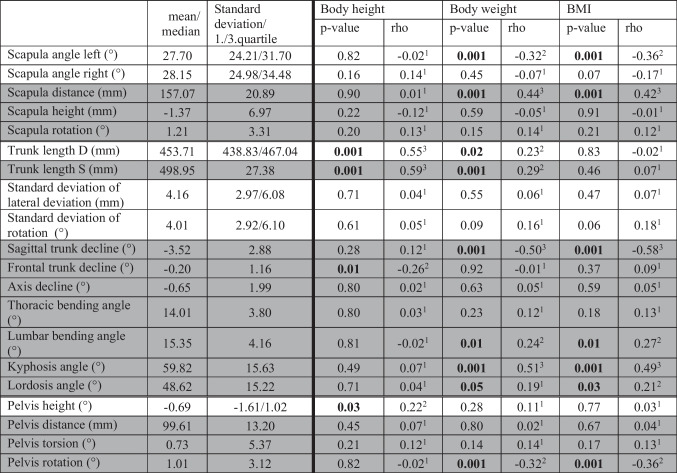
Mean/median, standard deviation/1 and 3 quartiles of the of the upper body posture as well as correlations between body height, weight, BMI, and back parameters. Normally distributed parameters are highlighted in gray. Significant *p*-values are marked in bold. Effect size according to Cohen (^1^: *r* = 0.1 low correlation, ^2^: *r* = 0.3 medium correlation, ^3^: *r* = 0.5 high correlation)

With higher weight and BMI, the test subjects are more forwardly inclined (sagittal trunk inclination, each *p* ≤ 0.001). In addition, the body weight and BMI correlate positively with the kyphosis angle (each *p* = 0.001), the lordosis angle (weight *p* ≤ 0.05, BMI *p* ≤ 0.03) and the lumbar bending angle (weight *p* ≤ 0.01, BMI *p* ≤ 0.001) (Table 1). There is also a positive correlation for the scapula distance (both weight and BMI *p* ≤ 0.001) and pelvic distance (weight *p* ≤ 0.001, BMI *p* ≤ 0.01) (Table 1).

In the BMI group comparison (Table 2), the mean shoulder distance (*p* = 0.001) increases with increasing group number. After the Bonferroni-Holm correction of the multiple Conover-Iman comparisons, only the significance between groups II and III (*p* = 0.02) and groups II and IV (*p* = 0.001) remains. An increasing series of negative values can also be seen in the median values of the sagittal trunk inclination (*p* = 0.001): − 0.60° in group I, − 2.49° in group II, − 3.79° in group III, and − 7.47° in group IV. After Bonferroni-Holm correction of the multiple Conover-Iman comparisons, the significances remain between groups II and III (*p* = 0.01), groups II and IV (*p* = 0.001), and groups III and IV (*p* = 0.02). The mean lumbar bending angle (*p* = 0.001), after applying the Bonferroni-Holm correction of the multiple Conover-Iman comparisons, shows significance only between groups II and III (*p* = 0.01) and between groups II and IV (*p* = 0.02). The kyphosis angle (*p* = 0.001) shows significance only between groups II and III (*p* = 0.001) and between groups II and IV (*p* = 0.01) after applying the Bonferroni-Holm correction of the multiple Conover-Iman comparisons. When examining the values of the lordosis angle (*p* = 0.03), only the median values of groups II (44.96°) and III (55.46°) are statistically significant (*p* = 0.02). For the pelvic distance DD (*p* = 0.01), significance remains between groups II and IV (*p* = 0.01) and between groups III and IV (*p* = 0.02) after Bonferroni-Holm correction of the multiple Conover-Iman comparisons.

**Table 2:**
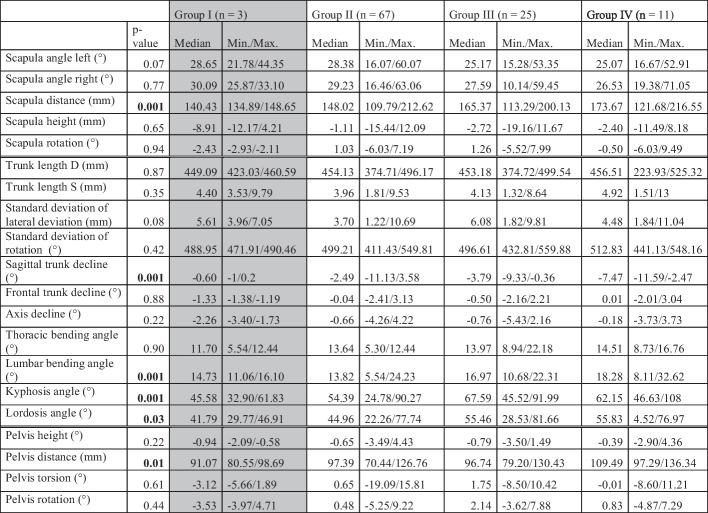
BMI group comparison of the test persons, divided into their respective body mass index group. Significant *p*-values are marked in bold. Due to the small number of cases, group I is only described descriptively and is not included in the statistical calculation, and is, therefore, highlighted in gray

### Orthodontic cast analysis

There are no significances (*p* ≥ 0.05) between the women with completed orthodontic treatment and those without treatment with regard to their upper body posture.

### Angle classes

When comparing the groups of the three different Angle classes (class I: *n* = 29, class II: *n* = 40, class III: *n* = 37), no statistically significant differences are found with regard to the back parameters (*p* ≥ 0.05).

### Midline shift in the mandible

Here, there are significant, positive correlations between axial deviation (*p* ≤ 0.05), pelvic position (*p* ≤ 0.01), standard deviation of lateral deviation (*p* ≤ 0.04), and rotation (*p* ≤ 0.02) as well as a significant, negative correlation of pelvic torsion (*p* ≤ 0.01) (Table 3). In the group comparison (group I: mandibular midline shift to the left (*n* = 26), group II: no midline shift (*n* = 57), group III: midline shift to the right (*n* = 23); Table 4), there are significances for the axial deviation (*p* ≤ 0.01) and pelvic position (*p* ≤ 0.01). The alignment of the upper body posture follows the direction of the midline shift, with a left-sided tendency. After Bonferroni-Holm correction of the multiple Conover-Iman comparisons, significances remain between groups I and II (*p* ≤ 0.01) and between groups I and III (*p* ≤ 0.02). In group I, the upper body of these participants tilts to the left by − 2.12°, while in group II (no MLS) this is − 0.30° and in group III (MLS to the right) it tilts only marginally to the left by − 0.28°. After Bonferroni − Holm correction, the pelvic level between groups I and II (*p* ≤ 0.01) and groups I and III (*p* ≤ 0.01) is significant. The pelvic level of group I has a median value of − 1.28° (left iliac crest cranial), while that of group II is − 0.54° and that of group III is 0.35° (right iliac crest cranial).

**Table 3:** Correlation between right and left molar occlusion, midline shift, vertical incisor step, and transverse width and back parameters. Significant *p*-values are marked in bold. Effect size according to Cohen (^1^: *r* = 0.1 low correlation, ^2^: *r* = 0.3 medium correlation, ^3^: *r* = 0.5 high correlation)

Parameter	Right molar occlusion		Left molar occlusion		Midline shift lower jaw		*Vertical incisor step*		*Transverse width*	
	*p*-value	Rho	*p*-value	Rho	*p*-value	Rho	*p*-value	Rho	*p*-value	Rho
Scapula angle left (°)	**0.03**	0.21^2^	0.90	− 0.01^1^	0.18	0.13^1^	**0.04**	− 0.20^2^	0.33	− 0.10^1^
Scapula angle right (°)	0.38	0.09^1^	0.17	− 0.14^1^	0.93	0.01^1^	0.16	− 0.14^1^	0.99	− 0.001^1^
Scapula distance (mm)	0.56	− 0.06^1^	0.94	0.01^1^	0.79	− 0.03^1^	0.21	0.12^1^	0.35	0.09^1^
Scapula height (mm)	0.97	0.001^1^	0.45	0.07^1^	0.91	0.01^1^	0.80	0.02^1^	0.10	− 0.16^1^
Scapula rotation (°)	0.99	− 0.001^1^	0.77	− 0.03^1^	0.20	0.13^1^	0.45	− 0.07^1^	0.55	0.06^1^
Trunk length D (mm)	0.09	0.17^1^	0.80	− 0.02^1^	0.86	− 0.12^1^	0.27	0.11^1^	**0.03**	− 0.22^2^
Trunk length S (mm)	0.17	− 0.14^1^	**0.01**	− 0.26^2^	**0.04**	0.20^2^	0.68	0.04^1^	0.39	0.08^1^
Standard deviation of lateral deviation (mm)	0.74	− 0.03^1^	0.63	− 0.05^1^	**0.02**	0.22^2^	0.42	− 0.08^1^	0.26	− 0.11^1^
Standard deviation of rotation (°)	0.12	0.15^1^	0.25	− 0.11^1^	0.95	0.01^1^	0.23	0.12^1^	0.21	− 0.12^1^
Sagittal trunk decline (°)	0.08	0.17^1^	0.08	0.17^1^	0.63	− 0.05^1^	0.43	0.08^1^	0.87	− 0.02^1^
Frontal trunk decline (°)	0.60	0.05^1^	0.63	− 0.05^1^	0.66	− 0.04^1^	0.41	− 0.08^1^	0.34	0.09^1^
Axis decline (°)	0.41	0.08^1^	**0.001**	− 0.28^2^	**0.05**	0.19^1^	0.60	0.05^1^	0.97	0.001^1^
Thoracic bending angle (°)	**0.01**	− 2.50^3^	0.24	− 0.12^1^	0.97	0.001^1^	0.55	− 0.06^1^	0.98	0.001^1^
Lumbar bending angle (°)	0.47	− 0.07^1^	0.53	0.06^1^	0.58	− 0.06^1^	0.79	− 0.03^1^	0.17	− 0.13^1^
Kyphosis angle (°)	0.21	− 0.12^1^	0.68	− 0.04^1^	0.97	0.001^1^	0.67	− 0.04^1^	0.30	− 0.10^1^
Lordosis angle (°)	0.20	− 0.12^1^	0.66	− 0.04^1^	0.75	− 0.03^1^	0.96	0.01^1^	0.58	− 0.05^1^
Pelvis height (°)	0.93	0.01^1^	**0.001**	− 0.28^2^	**0.01**	0.24^2^	0.38	0.09^1^	0.62	− 0.05^1^
Pelvis distance (mm)	0.55	0.06^1^	0.55	0.06^1^	0.45	0.07^1^	0.91	0.01^1^	0.56	− 0.06^1^
Pelvis torsion (°)	0.98	− 0.001^1^	0.89	0.01^1^	**0.01**	− 0.27^2^	**0.01**	0.26^2^	0.25	− 0.11^1^
Pelvis rotation (°)	0.50	− 0.07^1^	0.90	− 0.01^1^	0.51	0.06^1^	0.35	− 0.09^1^	0.60	0.05^1^

**Table 4:** Group comparison of the subjects, divided according to an existing midline shift in the mandible. Significant *p*-values after the Bonferroni-Holm correction are marked in bold

		Group 1 (*n* = 26) Midline shift to the left		Group 2 (*n* = 57) No midline shift		Group 3 (*n* = 23) Midline shift to the right	
	*p*-value	Median	Min./max	Median	Min./max	Median	Min./max
Scapula angle left (°)	0.94	28.25	16.40/53.33	27.63	15.28/60.07	27.51	18.10/54.93
Scapula angle right (°)	0.98	29.55	10.14/59.46	27.85	16.46/63.06	28.15	20.76/71.05
Scapula distance (mm)	0.79	158.69	126.27/195.35	156.79	109.79/216.55	160.42	121.68/212.62
Scapula height (mm)	0.90	− 1.49	− 15.4/9.08	− 0.81	− 13.93/11.68	− 2.23	− 19.16/12.09
Scapula rotation (°)	0.39	0.37	− 4.94/7.19	1.24	− 6.03/9.49	2.76	− 6.03/6.59
Trunk length D (mm)	0.70	453.24	410.95/488.86	453.66	374.72/525.32	455.77	223.93/496.17
Trunk length S (mm)	0.14	3.56	2.04/9.41	4.29	1.32/13	4.53	1.51/10.69
Standard deviation of lateral deviation (mm)	0.75	− 5.97	− 14.21/4.68	− 6.13	− 16.79/16.28	− 6.66	− 24.11/9.46
Standard deviation of rotation (°)	0.56	3.71	2.28/8.58	3.78	1.26/11.04	5.11	1.22/10.69
Sagittal trunk decline (°)	0.91	6.52	− 13.88/55.32	4.83	− 18.79/64.89	7.06	− 14.99/54.09
Frontal trunk decline (°)	0.84	496.58	441.13/547.81	500.29	432.81/559.88	500.55	411.43/549.81
Axis decline (°)	0.89	− 2.90	− 9.33/3.58	− 3.55	− 11.13/1.97	− 3.07	− 11.59/1.46
Thoracic bending angle (°)	0.14	− 0.47	− 2.16/2.21	0.02	− 2.41/3.13	− 0.11	− 2.39/1.21
Lumbar bending angle (°)	**0.01**	− 2.12	− 5.43/4.22	− 0.30	− 3.40/3.22	− 0.28	− 3.27/4.02
Kyphosis angle (°)	0.93	13.10	8.94/27.24	14.12	5.30/23.57	13.60	8.83/28.99
Lordosis angle (°)	0.94	15.51	8.49/23.07	14.65	8.11/32.62	14.81	5.54/22.31
Pelvis height (°)	1	58.83	31.95/91.62	60.92	32.90/108	57.21	24.78/91.99
Pelvis distance (mm)	0.66	48.99	22.26/81.66	45.92	5.88/77.74	49.19	4.52/77.47
Pelvis torsion (°)	**0.01**	− 1.28	− 3.45/3.22	− 0.54	− 3.49/3.70	0.35	− 3.50/4.43
Pelvis rotation (°)	0.60	97.99	70.44/128.42	97.63	75.49/130.43	103.09	75.22/136.34
Scapula angle left (°)	0.58	1.44	− 9/15.81	0.44	− 8.60/11.21	0.41	− 19.09/10.42
Scapula angle right (°)	0.33	0.10	− 4.39/7.88	1.78	− 4.87/9.22	0.60	− 5.25/6.16

### Horizontal and vertical anterior tooth steps

There are no significances between the gradations of the horizontal anterior tooth step and the parameters of the upper body posture.

With regard to the vertical anterior tooth level, a significance (*p* ≤ 0.01) between groups I (normal range; median 48.09°) and II (deep bite; median 60.92°) and between groups I and III (open bite; median 62.47°) with *p* ≤ 0.01 can be determined in the group comparison for the kyphosis angle and also for the scapula distance (*p* ≤ 0.001) (Table 5). The latter shows significance between groups I and II after Bonferroni-Holm correction (group I: normal range; median 141.01 mm; group II: deep bite; median 162.49 mm). The left shoulder stance angle (*p* ≤ 0.04) and pelvic torsion (*p* ≤ 0.01) show a significant correlation.

**Table 5:** Group comparison of the test subjects, divided according to their vertical anterior tooth stage. Significant *p*-values are printed in bold type

		Group 1 (*n* = 17) Norm		Group 2 (*n* = 72) Deep bite		Group 3 (*n* = 17) Tendency to open bite/open bite	
	*p*-value	Median	Min./max	Median	Min./max	Median	Min./max
Scapula angle left (°)	0.07	30.53	23.18/53.35	26.73	15.28/60.07	28.65	19.18/33.46
Scapula angle right (°)	0.17	32.25	17.06/69.50	27.55	10.14/63.06	30.74	18.05/71.05
Scapula distance (mm)	**0.001**	141.01	113.29/198.33	162.49	109.79/216.55	148.65	126.27/185.76
Scapula height (mm)	0.60	− 3.11	− 13.68/9.20	− 0.96	− 19.16/12.09	− 2.18	− 9.61/8.18
Scapula rotation (°)	0.73	1.35	− 2.46/7.99	0.69	− 6.03/9.49	2.23	− 6.03/6.47
Trunk length D (mm)	0.42	449.21	223.93/488.86	455.72	397.10/507.11	449.07	374.71/525.32
Trunk length S (mm)	0.92	3.88	1.51/10.69	4.03	1.32/13	4.26	1.96/8.64
Standard deviation of lateral deviation (mm)	0.73	3.70	1.80/9.81	4.01	1.22/11.04	4.36	1.94/8.37
Standard deviation of rotation (°)	0.77	497.18	435.56/548.86	500.29	441.13/549.81	493.2	411.43/559.88
Sagittal trunk decline (°)	0.46	− 3.57	− 11.59/0.54	− 2.90	− 10.30/3.58	− 3.79	− 11.13/1.57
Frontal trunk decline (°)	0.82	− 0.11	− 2.23/1.21	− 0.19	− 2.41/3.13	0.02	− 2.02/2.12
Axis decline (°)	0.25	− 0.18	− 2.73/3.73	− 1.12	− 5.43/4.22	− 0.76	− 2.63/1.41
Thoracic bending angle (°)	**0.04**	12.90	9.60/17.94	13.84	5.30/27.24	16.14	11.70/28.99
Lumbar bending angle (°)	0.14	13.60	8.84/20.03	15.49	5.54/32.62	15.79	11.06/23.07
Kyphosis angle (°)	**0.001**	48.09	34.85/66.55	60.92	24.78/108	62.47	35.48/90.27
Lordosis angle (°)	0.79	44.36	4.52/71.48	48.92	21.66/81.66	51.41	5.88/72.50
Pelvis height (°)	0.21	0.33	− 2.01/4.36	− 0.73	− 3.50/4.43	− 0.78	− 2.21/1.49
Pelvis distance (mm)	0.26	93.46	77.18/136.34	98.79	70.44/128.42	104.28	75.22/130.43
Pelvis torsion (°)	0.18	− 0.33	− 7.19/9.86	1.21	− 19.09/15.81	0.82	− 8.60/5.72
Pelvis rotation (°)	0.93	0.81	− 4.15/6.38	0.51	− 5.25/7.88	1.78	− 3.62/9.22

### Transverse width of the maxillae and mandibles

The transversal width of the upper jaw shows a negative significant correlation with the trunk length D (*p* ≤ 0.03). The evaluation of the data regarding the correlations between the transversal width of the maxillae and mandibles and upper body posture in the transversal direction does not reveal any significances.

### Right- and left-sided molar occlusions

A right-sided mesial occlusion can be detected in 20 female subjects (18.87%). The majority show a neutral (*n* = 41 women, 38.68%) or distal occlusion (*n* = 45 women, 42.45%) on the right side. Twenty-nine female subjects show mesial toothing on the left side (27.36%), while 45 women show a neutral (42.45%) and 32 a distal occlusion (30.19%) on the left side.

The right-sided molar occlusion correlates significantly with the left shoulder stance angle (*p* ≤ 0.03) and the thoracic bending angle (*p* ≤ 0.01). The more mesial the right-sided occlusion, the more caudal the left scapula become, and the smaller the value of the thoracic kyphosis. With regard to the left-sided occlusion, significant correlations are found in the standard deviation of the lateral deviation (*p* ≤ 0.01), the axial deviation (*p* ≤ 0.001), and the pelvic position (*p* ≤ 0.001). The more mesial the left-sided occlusion, the lower the tilt between the upper body and the pelvis, the more left-sided the upper body lateral tilt, and the higher the left pelvis stands can be observed.

### Axiography

The greater the protrusion movement of the mandible, the smaller the lumbar bending (*p* ≤ 0.03) and kyphosis angle (*p* ≤ 0.03), and the further the upper body is inclined dorsally (up to 4°) (*p* ≤ 0.04; Table 6). When comparing the subjects with a protrusion in the norm and those with an enlarged protrusion, the lordosis angle is significant (*p* ≤ 0.001); this angle is larger for subjects with a norm protrusion (52.34°) than those with an enlarged protrusion (41.79°).

**Table 6:** Correlation between protrusion and back parameters. Significant *p*-values are marked in bold. Effect size according to Cohen (^1^: *r* = 0.1 low correlation, ^2^: *r* = 0.3 medium correlation, ^3^: *r* = 0.5 high correlation)

	Protrusion		Laterotrusion right		Laterotrusion left		Mouth opening	
Parameter	*p*-value	Rho	*p*-value	Rho	*p*-value	Rho	*p*-value	Rho
Scapula angle left (°)	0.23	0.12^1^	0.07	0.17^1^	0.42	0.08^1^	0.99	0.001^1^
Scapula angle right (°)	0.33	0.10^1^	**0.02**	0.23^2^	0.15	0.14^1^	0.64	0.05^1^
Scapula distance (mm)	0.48	0.07^1^	0.89	− 0.01^1^	0.91	− 0.01^1^	**0.04**	0.19^1^
Scapula height (mm)	0.57	0.06^1^	0.52	− 0.06^1^	0.66	0.04^1^	0.29	0.10^1^
Scapula rotation (°)	0.71	− 0.04^1^	0.44	0.08^1^	0.30	0.10^1^	0.11	0.16^1^
Trunk length D (mm)	0.76	0.03^1^	0.18	0.13^1^	0.93	− 0.01^1^	**0.05**	0.19^1^
Trunk length S (mm)	0.65	− 0.04^1^	0.30	− 0.10^1^	0.65	− 0.04^1^	0.18	− 0.13^1^
Standard deviation of lateral deviation (mm)	0.82	− 0.02^1^	0.32	− 0.15^1^	0.78	− 0.03^1^	0.41	0.08^1^
Standard deviation of rotation (°)	0.54	0.06^1^	0.41	0.08^1^	0.75	− 0.03^1^	**0.03**	0.21^2^
Sagittal trunk decline (°)	**0.04**	0.20^2^	0.71	0.04^1^	0.25	0.11^1^	0.26	− 0.11^1^
Frontal trunk decline (°)	0.16	− 0.14^1^	0.32	0.10^1^	0.69	0.04^1^	0.98	− 0.001^1^
Axis decline (°)	0.50	− 0.07^1^	0.23	0.12^1^	0.75	− 0.03^1^	0.22	0.12^1^
Thoracic bending angle (°)	0.43	− 0.08^1^	0.31	− 0.10^1^	0.24	− 0.11^1^	0.10	0.16^1^
Lumbar bending angle (°)	**0.03**	− 0.22^2^	0.91	− 0.01^1^	0.80	− 0.03^1^	0.54	− 0.06^1^
Kyphosis angle (°)	**0.03**	− 0.21^2^	0.25	− 0.11^1^	0.06	− 0.18^1^	0.52	0.06^1^
Lordosis angle (°)	0.07	− 0.18^1^	0.98	− 0.001^1^	0.12	− 0.15^1^	0.99	− 0.001^1^
Pelvis height (°)	0.95	− 0.01^1^	0.53	0.06^1^	0.51	− 0.06^1^	0.14	0.15^1^
Pelvis distance (mm)	0.91	− 0.01^1^	0.85	0.02^1^	0.72	− 0.04^1^	0.19	0.13^1^
Pelvis torsion (°)	0.44	− 0.08^1^	0.82	− 0.02^1^	0.86	− 0.02^1^	0.81	0.02^1^
Pelvis rotation (°)	0.83	− 0.02^1^	0.44	0.08^1^	0.34	0.09^1^	0.06	0.18^1^

There is a significant positive correlation between the laterotrusion to the right and the right shoulder stance angle (*p* ≤ 0.02). No correlations can be found between laterotrusion to the left, or deviations and deflections, as well as the maximum mouth opening, and changes in the upper body statics (*p* ≥ 0.05).

## Discussion

The present study with 106 healthy women aged 31–40 years aimed to analyze correlations between occlusions and the upper body posture using medical history data, cast analysis, axiography, and video raster stereography. With a mean height of 1.66 m, a mean body weight of 67.15 kg, and a mean BMI of 24.25 kg/m^2^, these constitutional data are comparable to existing studies [44, 45]. The statistical analysis of these parameters proves a positive correlation between the ventral tilt of the trunk and weight or BMI, which is confirmed by the BMI group comparison (group II: − 2.49°, group III: − 3.79°, group IV: − 7.47°). The kyphosis and lordosis angles as well as the lumbar bending angle increase with increasing weight and BMI..

It is possible that the increased body weight causes pressure on the spine which, thus, also influences the spinal balance due to the altered lordosis and kyphosis. This is based on the theory that the sagittal alignment of the spine is primarily stabilized by the balance between lumbar lordosis, thoracic kyphosis, and the pelvic position [46]. This is accompanied by energy-consuming compensatory mechanisms in the spine, pelvis, and lower extremities. In a study by Jankowicz-Szymanska et al. [47] of 271 girls and 241 boys aged 10–12 years, measuring these sagittal parameters with an ultrasound system and using a platform to measure the arch of the foot showed a positive correlation between BMI and the reduced arch of the foot and a more pronounced lumbar lordosis.

In obese individuals, body mass gain is accompanied by increasing hyperkyphosis and an anteriorly shifted center of gravity [24, 25, 48]. An increased mechanical load on the lumbar spine with a resulting increase in the lordosis angle due to increased weight has also been demonstrated by Onyemaechi et al. [49] using lateral radiographs. Lumbar lordosis is strongly determined by sacral alignment and acts as a compensatory element of kyphosis [50]. Degenerative changes and narrowing of the intervertebral discs also exacerbates physiological thoracic kyphosis [51]. These skeletal changes with an accompanying increase in kyphosis are not assumed to occur in the subject population of this study as, according to Fon et al. [31], they only occur after the age of 40 years. However, breast size may be associated with increasing BMI and increased thoracic kyphosis [26] and, therefore, could be an explanation for the observed increased trunk tilt [52]. A higher BMI is associated with more body mass; this increased fat or muscle tissue causes a wider scapula and pelvic distance and may, thus, also affect the shoulder stance angle. The results of the present study can be confirmed, among others, in female participants between 21 and 30 years and in male adults in the age groups of 31–40 years and 51–60 years, but not in female adults between 51 and 60 years [23]. However, the latter group of subjects had a higher proportion of underweight and also a lower proportion of obese women than in the present study.

The sagittal occlusion deviation, differentiated into Angle classes I, II, and III, is not reflected in the relevant parameters to the sagittal plane, i.e., the sagittal trunk inclination, lumbar bending angle, kyphosis angle, as well as the lordosis angle. The deviations of the upper body posture of the subjects with Angle classes II and III malocclusions are only minimal from those with Angle class I. Possible one-sided Angle classes could also have an influence here; however, these were not differentiated in this study but should be considered in the future. Thus, the available data cannot confirm the correlations found between Angle class II or II and the anteriorly/posteriorly posture by Gadotti et al. [13] and Nobili et al. [14].

The results of Lippold et al. [11, 12] and Solow et al. [10] cannot be confirmed either. However, cephalometric images were used in these two studies and not in the present study. In Germany, it is not ethically justifiable to take cephalometric images of healthy people without an indication for therapy, and therefore, the model analysis in the present study was carried out without this information. It must also be considered that present subjects were adult, healthy women aged 31–40 years and not children or adolescents as in the studies of Lippold et al. [12] or Klostermann et al. [13]. In the latter investigation, however, the significant results are so low that they lie within or just outside the measurement error range of video raster stereography.

Considering the fact that despite the high number of orthodontic treatments performed (40.57%), there is a large collective of Angle class II (37.74%) and III (34.90%) and, thus, it can be assumed that the study participants have a stable musculoskeletal system which, with its overall stability, can compensate for recurrence. Therefore, the dental deviation did not transfer to the body as a possible recurrence following orthodontic treatment. This is accompanied by the fact that there is no difference in the upper body posture between orthodontically treated and untreated subjects.

Furthermore, it is striking that both on the right and on the left side, a mesially oriented occlusion shows a leftward inclination of the frontal pelvic tilt, namely the further mesially the left molars are positioned. In addition, a right-sided mesial occlusion shows a more caudal left scapula. A tendency towards conspicuousness of the left side can be identified which should be analyzed in more detail in further studies; it can be assumed that the laterality of the body plays a role here. The dominant right side of the mainly right-handed test persons (*n* = 102) seems to be able to compensate for a change in occlusion without affecting the upper body.

The vertical overbite shows that the smaller the overbite, the more caudal the left shoulder stance angle becomes. Furthermore, the vertical anterior group in the normal range showed a kyphosis angle of 48.09°, with a deep bite of 60.92° and an open bite of 62.47°. The group in the normal range deviates significantly from the other two. As the stable occlusal support provided by a regular overbite is lacking having a vertical overbite above or below the normal range, the head may be tilted anteriorly resulting in a more kyphotic thoracic posture. The same is the consequence of a deep overbite.

Breithecker [53] assumes that head posture is unconsciously influenced by the occlusion of the teeth, so that the posture of the body can be influenced by the position of the head. Alkofide et al. [27] also detected a significance in the craniocervical and cervicohorizontal angle between subjects with and without a deep bite. Lopatiene et al. [28] also confirmed the correlation between a deep bite and scapula asymmetries in adolescents.

With regard to the axiography, it can be seen that with increasing protrusion, the ventral inclination of the upper body is reduced (up to 4° dorsally). At the same time, the smaller the lumbar bending angle and the kyphosis angle, the greater the protrusion movement of the mandible results. In addition, participants with an increased mandibular advancement movement show an approximately 9° (norm: 52.34°, > norm: 41.79°) lower lordosis angle. The spinal curvatures are, thus, less pronounced and, therefore, the more upright stance allows more lower jaw movement in the sense of protrusion. Accordingly, there is a correlation between the extent of mandibular movement and upper body posture in the sagittal direction but not in the frontal plane. Zhou et al. [29], on the other hand, found a change in the shoulder angle range linear to the deviation of the mandibular deformity in mandibular deviation. Thus, a skeletal component of deviation seems to exert a greater influence on posture than a myogenic influence in the present study, which is suggested by the high number of cases (88.68%). Even muscular tension in the head/neck/shoulder area or a highly stressed masticatory musculature, e.g., due to nocturnal grinding/pressing, can alter or limit the movement recording of the axiograph, which, however, does not represent a disease value as there were no pain symptoms in the subjects. The anatomical condition of the masticatory muscles and the fatty tissue also play a role.

With regard to limitations, it should be noted that the group size of the BMI was not evenly distributed as most of the test subjects were in the normal range according to the WHO [36]. Although, this goes in line with other findings, so that the BMI should be analyzed in a more differentiated way.

In summary, it can be said that especially not only the constitutional parameters (BMI, weight), but also the protrusion or midline shift, have an influence on the upper body posture. Changes mainly occur in the sagittal plane (spinal curvatures, upper body displacement), while the midline shift has only effects in the frontal plane. The constitutional parameters have effects in both planes.

## Conclusion

The examination of 106 subjectively healthy women aged 31–40 years showed positive correlations of the lumbar bending angle and the kyphosis and lordosis angles with increasing weight as well as a stronger ventral inclination of the trunk with increasing weight and BMI. Regarding the dental parameters of the cast analysis, a left lateral tilt of the trunk was evident, regardless of the direction of the midline shift. Despite a small difference in the values, the frontal pelvic tilt and the side of the displacement of the jaw correlated. It should be noted that the left side was more pronounced. The presence of an open or deep bite was also associated with a more pronounced kyphosis angle. The greater the protrusion, the smaller the sagittal plane angle (kyphosis angle, lumbar bending angle), and the greater the sagittal torso inclination resulted.

## Data Availability

All relevant data are shown in the manuscript.
